# The *INSIGHT* project: reflections on the co-production of a quality recognition programme to showcase excellence in public involvement in health and care research

**DOI:** 10.1186/s40900-023-00508-4

**Published:** 2023-10-25

**Authors:** Steven Blackburn, Rachele Hine, Samantha Fairbanks, Phillip Parkes, Darren Murinas, Andrew Meakin, Robert Taylor, Linda Parton, Marilyn Jones, Jessica Tunmore, Jennifer Lench, Nicola Evans, Katharine Lewney, Lucy O’Mara, Anthony A. Fryer

**Affiliations:** 1 Institute for Applied Health Research, University of Birmingham, Birmingham, UK; 2 Expert Citizens CIC, The Dudson Centre, Hanley, Staffordshire, UK; 3 Research User Group, Keele University, Keele, Staffordshire, UK; 4 Expert by Experience, Shropshire, UK; 5 Research and Innovation Department, Midlands Partnership University NHS Foundation Trust, St George’s Hospital, Stafford, UK; 6 Impact Accelerator Unit, School of Medicine, Keele University, Keele, Staffordshire, ST5 5BG UK; 7 Directorate of Research and Innovation and Centre for NMAHP Research and Education Excellence (CeNREE), University Hospitals of North Midlands, Staffordshire, UK

**Keywords:** Public involvement, Quality improvement, Appreciative inquiry, Co-production, UK Standards for Public Involvement

## Abstract

**Background:**

The quality of Patient and Public Involvement (PPI) in healthcare research varies considerably and is frequently tokenistic. We aimed to co-produce the Insight | Public Involvement Quality Recognition and Awards programme, based on the UK Standards for Public Involvement (UKSPI) alongside an incremental scale designed by Expert Citizens (a lived experience-led community group), to incentivise and celebrate continuous improvement in PPI.

**Methods:**

We used Task and Finish Groups (19/44 [43%] public contributor membership) to co-produce the programme which we piloted in three organisations with different healthcare research models. We used surveys and review sessions to capture learning and reflections.

**Results:**

We co-created:

1. A Quality descriptor matrix comprising four incremental quality levels (Welcoming, Listening, Learning, Leading) for each UKSPI standard.
2. An assessment framework including guidance materials, self-assessment form and final report template.
3. An assessor training package.
4. The quality awards event format and nomination form. These materials were modified based on pilot-site feedback.

Of survey respondents: 94.4% felt they had made at least ‘*Some’* personal contribution (half said ‘*Quite a lot*’/‘*A great deal’*), 88.9% said they were ‘*Always’/*‘*Often’* able to express their views freely and, 100% stated the programme would have ‘*A lot of impact*’/‘*Quite a bit of impact*’.

During the project, we identified the importance of taking time to explain project aims and contributor roles, adapting to the needs of individual contributors and, using smaller bespoke sessions outside the main Task and Finish Groups.

**Conclusions:**

We co-produced and piloted a quality recognition programme to incentivise and celebrate continuous quality improvement in PPI. One public contributor stated, “*I feel strongly that the Insight framework and awards will raise awareness of the* [public involvement] *work going on in many community settings.* [It] *is likely to result in better sharing of positive practice, incentivising research groups of any size to start work or to improve the quality of* [PPI] *could be one of the main benefits. I’m excited that if this initiative takes off, regionally and then in the longer term nationally, it could be a significant step in advancing the* [public]* voice.”*

**Supplementary Information:**

The online version contains supplementary material available at 10.1186/s40900-023-00508-4.

## Abstract (plain language summary)

How researchers involve members of the public in health research varies widely. We developed a scheme that encourages researchers from any health research organisation to improve the quality of public involvement. We used joint workshops with researchers, health workers and members of the public to design the scheme. We then tested it in three research organisations. We recorded the experience of people taking part in the project to learn what went well and what could be improved. We looked at the six areas covered in the UK Standards for Public Involvement. For each area, we worked together to define four levels (Welcoming, Listening, Learning, Leading) of increasing quality. We designed the materials needed for organisations to take part in the scheme. We also created a training pack for assessors and the format of a celebration event. We modified the materials after testing them. We asked those who took part in the project, half of whom were lay members, what they thought. 94% felt they had made at least some personal contribution. 89% said they were often or always able to express their views freely. Everyone thought the project would have some degree of impact. Overall, those members of the public who took part said they enjoyed the process and felt that their views were listened to. Along the way, we learned that it was important to carefully explain the project’s aims, be clear about roles and have 1to1 discussions outside the main workshops.

**Supplementary Information:**

The online version contains supplementary material available at 10.1186/s40900-023-00508-4.

## Background

### The importance of public involvement

Involving those with ‘lived experience’ in the development and delivery of healthcare research (frequently referred to as Patient and Public Involvement; PPI)—including patients, carers and members of the wider public—is scientifically and ethically essential [1–3].

In practice, this means that PPI should be part of the entire journey of a research project; from initial conception, project design, project oversight, participant recruitment, logistics, data collection, interpretation and dissemination of results and indeed leadership [4–6]. However, public involvement also has wider applications, such as in formulating strategic direction at local and national level, departmental- or organisational-level oversight and review of funding bids [2, 4, 7].

### Incentivising public involvement

While this involvement of the public in health and social care research should be self-evident, there has been a tendency for researchers to drive the agenda without involving those who will be affected by the research [8]. It is sometimes seen as an academic exercise driven by performance pressures in academia and guidance for completing research funding applications, rather than as a route to higher quality, clinically-relevant research that meets the actual needs of patients [8, 9].

Accordingly, incentivising good quality PPI in healthcare research is critical and a number of initiatives have sought to support greater use of PPI. Furthermore, evidence of appropriate and active public involvement throughout a project is becoming an increasingly important requirement for research ethics applications [10] many funding bodies including the UK National Institute for Health and Care Research (NIHR) [11, 12], though approaches to PPI appear variable across different funding bodies [13].

To encourage improvements in PPI quality, the NIHR Centre for Engagement and Dissemination (CED) was set up to ‘*champion the effective engagement and involvement of patients, public, carers, service users and communities (people and communities) in all parts of the research journey’* [14]. Through the Public Involvement Standards Development Partnership, a set of standards were developed to clearly set out what effective public involvement looks like [15, 16]. These UK Standards for Public Involvement (UKSPI) in research were designed to encourage reflection and learning as a means to improving PPI rather than a set of hard indicators to evaluate PPI performance. However, they provide a very valuable and adaptable framework against which PPI practice can be recognised and improvement opportunities reflected upon.

### The gap

While several other PPI incentivisation schemes exist, these are: restricted to small teams/individuals [17–19], focus on specific sector (e.g. academia) [20], have an emphasis on public engagement rather than involvement [20], are more like an audit/accreditation scheme [20], limited to specific organisations [21, 22], restricted to grants from specific funding bodies [23] or make no reference to improving the voice of seldom-heard communities [17–23]. Furthermore, what the UKSPI do not explicitly provide, and were not intended to, is an indication of *levels* of quality, nor do they specifically recognise or reward high quality PPI initiatives. Hence there is a need for a national scheme that incentivises high quality PPI by recognising, celebrating and sharing best practice. Our Research User Group members commented that, within their networks, the need for such incentivisation has been widely noted by public contributors. An incentivisation scheme would demonstrate that organisations value and respect public contribution, as well as mitigate the dangers of a ‘tick-box’ PPI culture [8, 9]. Any such scheme would need to address the current disincentives to participating in PPI (principally time and cost [1, 3, 24]).

### An exemplar

Independently of the UKSPI, Expert Citizens, a Community Interest Company based in Stoke-on-Trent, UK, developed a framework to rate the quality of involvement of people with lived experience for organisations providing services for people experiencing social disadvantage (e.g. homelessness, addiction, offending behaviour, mental health challenges). They comprise a team with lived experience themselves and therefore provide unique insight into the needs of services in this sector.

The Expert Citizens team developed the Insight Evaluation© programme and linked National Insight Awards [25, 26], which aims to recognise, celebrate and share positive practice regarding involving those with lived experience in service improvement. Using a co-production model, Expert Citizens created a framework comprising their own set of standards, each with four incremental quality levels: *Welcoming, Listening, Learning* and *Leading*. Importantly, they used an appreciative inquiry, strengths-based approach based on unconditional positive regard [27]. It was intentionally aimed at encouraging co-production in service design, development and delivery, rather than as an audit or accreditation tool to evaluate performance. Expert Citizens developed the original Insight standards in response to the question, “Who decides what positive practice looks like?” As such, the standards are an example of a disruptive systems change. They challenge underlying assumptions about the sources of knowledge and expertise to recognise and leverage lived experience as a source of power.

Although the original framework was not developed specifically for a healthcare context, there are several examples from previous award winners and nominees drawn from healthcare settings [28]. These examples illustrate that their Insight model has the capability of encouraging co-production in service design. 

#### What we set out to do:

Given the parallels between the aims of the UKSPI and Expert Citizens Insight Evaluation model, we explored the possibility of integrating the strengths of both approaches. Using these as a basis, we co-produced, together with the Expert Citizens team, what came to be known as the Insight | Public Involvement Quality Recognition and Awards Programme. As with the Expert Citizens model, this Programme comprised both a Quality Recognition Scheme and a National Quality Awards Event. Early in the co-production process (feedback from the launch event and early task and finish group sessions), we established that such a scheme would need to:

1. Be applicable across a wide variety of health and social care settings, from small community-based organisations to large NHS, academic or private sector institutions.
2. Genuinely involve those with lived experience in its creation and delivery (true co-production).
3. Be based on the same appreciative inquiry approach used by Expert Citizens to facilitate recognition and sharing of best practice, rather than as an audit or accreditation tool.

Our aims were therefore to co-create the framework for the Programme, using task and finish groups, and pilot the Programme across three organisations representing differing models of, and approaches to, healthcare service provision and research. We now describe the set-up, learning and outputs from this project. We also include the challenges faced along the way (and how these were addressed), and learning and reflections from both public and professional contributors on the co-production process. This project describes the first phase of a larger programme of work, which has now also included independent market research and the development of a business model, as well as work to develop the infrastructure to support the programme’s scalability and long-term sustainability (beyond the scope of this report).

## Methods

**Fig. 1:**
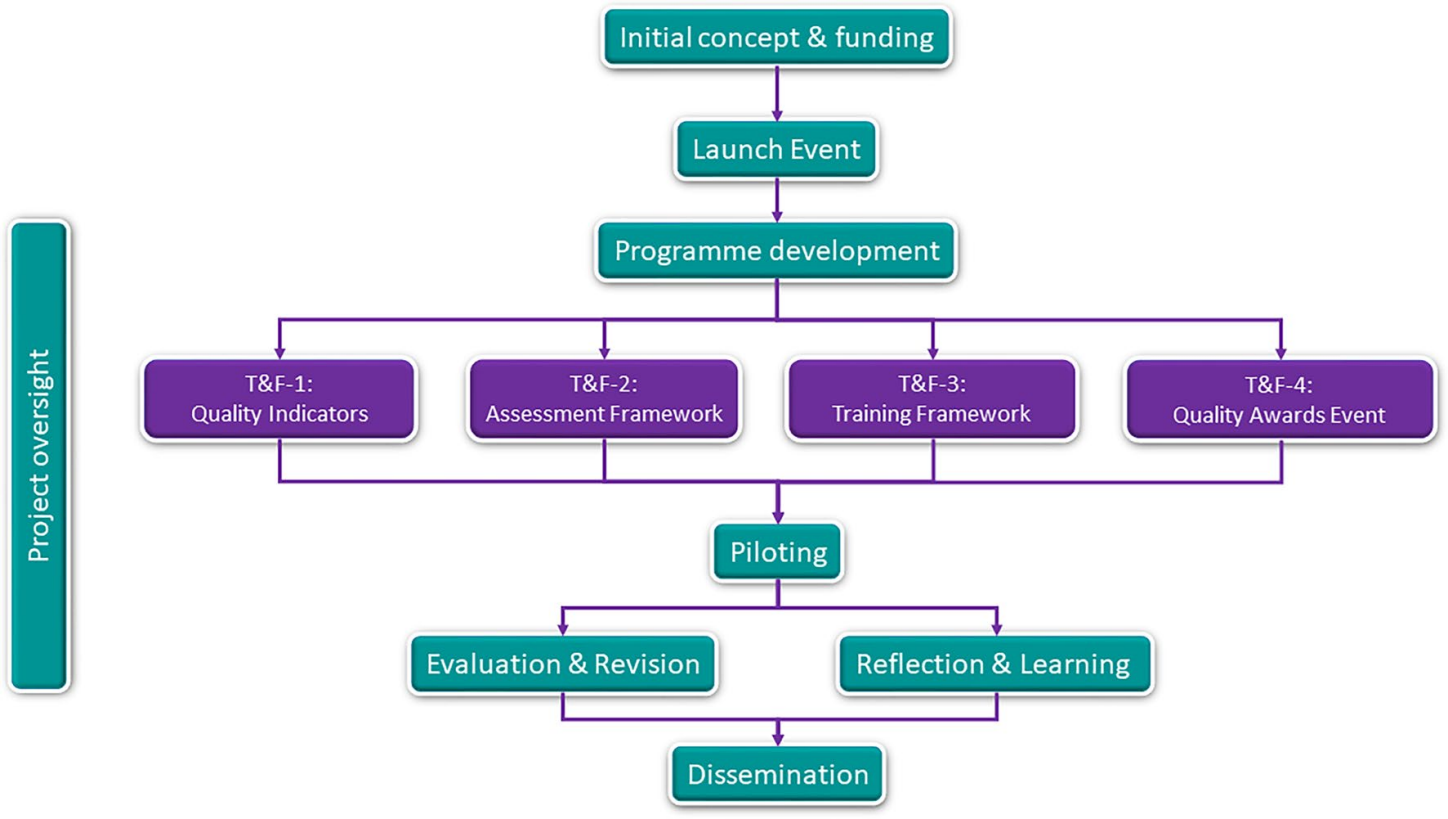
Flow chart showing project stages

The project involved a number of stages as outlined below (Fig. 1). Public contributors were involved at each stage (see Additional file 1—GRIPP2 Short Form). As this work was a PPI co-production project with public contributors as collaborators rather than data sources [29], research ethics committee approval was not required. However, the project did adhere to the general ethical principles outlined by UK Research and Innovation and by the Helsinki Declaration [30, 31]. Written consent was obtained to use quotes from public contributors in this publication. All public contributors were offered payment in line with National Institute for Health and Care Research recommendations [32]. Details of how the initial concept was conceived, project oversight arrangements and a description of the project launch event are provided in Additional File 2.

### Programme development

To develop the framework and key elements of the Programme, four ‘task and finish groups’ (TFGs) were created (led by AAF or SB, with a nominated scribe for each session [divided between NE and two other staff members]), each addressing one of the four elements of the Programme:

1. Quality Indicators: Establishment of the overall framework for the programme and development of the quality indicators based on the Expert Citizens Insight Evaluation Programme and the UKSPI.
2. Assessment: Creation of the Programme’s assessment framework.
3. Training: Development of a training package for assessor (both professional and lay assessors).
4. Quality Awards Event: Exploration of the logistics, format and assessment process for a national annual quality awards event.

Our initial plan was to use face-to-face meetings for the TFGs. However, as a result of the COVID-19 pandemic-associated restrictions, this was switched to an online platform (MS Teams). Care was taken to ensure that contributors felt comfortable participating in an online environment by providing support and guidance (e.g. a digital engagement guide [33]) and recognised best practice [34].

Co-production was central to the purpose of these groups (Additional file 1) [35, 36]. Membership comprised a broadly equal number of professional and public participants (19/44; 43%) from a variety of different backgrounds and experiences (21 TFG sessions over 33 weeks; Additional file 3), including those from Keele University’s Research User Group, Expert Citizens, and those affiliated with the other two partner organisations; Midlands Partnership Foundation NHS Trust and the University Hospitals of North Midlands NHS Trust. At the outset of each TFG, the roles and responsibilities of attendees and principles of co-production were agreed (based on NIHR principles [35]). In some instances, smaller group or 1:1 meetings were arranged alongside the TFGs for members to address specific tasks from the TFGs.

The aim of the TFGs was to create a working framework for the programme, along with associated documentation, to enable it to be piloted. The process for each TFG generally involved an initial wide discussion around a number of theme-specific topics, development of draft documentation, followed by review of the documentation by the group. For example, one of the documents that TFG 1 co-created was a description of the newly generated Quality Indicators which would then be used as a basis for the assessment and training covered by TFGs 2 and 3. 'Feedback was sought from TFG members and where appropriate the documents were further revised prior to the piloting of the programme.

### Piloting

The Programme was piloted at three sites. These were selected to gain feedback from different types of organisations at differing stages in their PPI journey; a multi-site acute university hospital NHS trust (University Hospitals of North Midlands Research and Innovation Directorate), a community NHS trust (Midlands Partnership Foundation University NHS Trust Research and Development Department) and a university department with ongoing clinical research (Keele University School of Pharmacy and Bioengineering).

During the piloting, we asked the sites for feedback on the documents and forms (e.g. structure, ease of completion, possible amendments), the format of the scheme (e.g. process, structure) and on its impact (e.g. overall value, changes made as a result of participation). We also encouraged pilot sites to suggest nominees for the Quality Awards Event to gain feedback on that process, including the completion of the newly-developed nomination form. The training package was also piloted with three new public assessors invited by an email invitation to members of the Keele University Research Users Group. AAF delivered the training virtually (via MS Teams) using the co-produced slides and took notes of suggested changes.

### Evaluation and revision

Following the pilots, AAF and SB obtained feedback from:

1. The TFG members to gain insight into how well the co-production process had worked for them (see below).
2. Pilot site participants to highlight any changes needed to the documentation and to explore the benefits of participation in the Programme.
3. Assessors to explore both public and professional experience of applying the new Quality Indicators, use of the documentation and completion of the final report. From new assessors, we were also able to gain views on the training package. Feedback was collected as part of meetings (e.g. from participants during the assessment process, or from assessors as part of the training itself) and via email from pilot sites.

### Reflection and learning

The development of INSIGHT drew on a range of reflection typologies that follow the principles of the model provided by Schön where the design process is, of itself, a reflective practice [37, 38]. The interactions between the project participants, including the task and finish groups, provided opportunities for ‘reflection-in-action’ leading to evolutionary changes in the emerging design of the framework. Subsequently, the piloting and end of project evaluation provided opportunities for ‘reflection-in-action’ that influenced the final form of the phase 1 outputs and helped to frame activity for later phases of the project. Both categories included elements of primarily experiential and narrative reflection among the participants.

Throughout the process, the scribes collected and AAF/SB collated the experiences and reflections of both public and professional contributors on the T&F process. As part of a review of meeting notes from the task and finish groups and final review session by the core team (SB, AAF, NE, RH, JT), how the input from public contributors shaped the final product throughout its development was captured. In addition, this review identified the learning garnered during the co-production journey, exploring what went well and how we might have done things differently, including any unexpected outcomes encountered along the way.

This was achieved using:

1. A simple bespoke online feedback questionnaire circulated to all those who participated in the TFG work, both professional and public (Additional file 4). This was developed by SB and AAF to capture a broad perspective on how participants felt about their involvement. It was based on a similar questionnaire used by Keele University for the JIGSAW-E project [39]. Replies were received from 18/44 (41%).
2. A review session (led by AAF) for all TFG participants at the end of the programme development phase
3. Ongoing discussions as part of the range of oversight meetings (see Additional file 2), TFG meetings, during piloting phase and document review meetings.
4. A request for written feedback from pilot sites on the format, content and potential impact of the final report.

Data on the number of public contributors involved in each TFG (Table 1) and in other elements of the project (Additional files 1, 2) was collated by NE.

**Table 1:** Structure and outputs of the Task and Finish groups

Task and finish group	1. Quality indicators	2. Assessment framework	3. Training framework	4. Quality awards event
Aims	Establishment of the overall programme structure and development of the quality indicators	Creation of the assessment framework	Development of an assessor training framework	Creation of the structure and assessment of the logistics of a quality awards event
Topics	What is already available?	What is meant by an assessment?	What does the assessor role look like?	What does the structure of the awards event look like?
	Who is the target audience?	What evidence would be required?	What kind of documents might assessors need to evaluate?	How would the nomination process work?
	What will the structure of the programme look like?	How would the evidence be collected?	What training would assessors need? How might this be delivered and by whom?	How would nominations be assessed and by whom?
	What are the descriptors for the quality indicators?	What would the expectations be for each of the four quality levels?	What would an assessment panel look like and where would we recruit them from?	What are the logistical and financial considerations for the awards event?
Membership (professional:public contributors)	8: 5	9: 7	5: 2	3: 5
Number of meetings	6	6	6	4
Outputs	*QI1. Quality Indicators*	*AF1. Process Map for Quality Recognition Scheme*	*TF1. Assessor role descriptors*	*QAE1. Process Map for Quality Awards Event*
	*QI2. Mind Map*	*AF2. Scheme Introduction Pack for Participating Organisations*	*TF2. Assessor Training Pack*	*TF2. Content of Training Pack regarding Quality Awards Event*

As expected (and encouraged) during the project, a diverse range of views and comments were received, including some instances where comments were mutually exclusive, and discussions became more lively and interactive as the project progressed and participants became more comfortable with each other. Generally, these were resolved and consensus achieved during the TFG discussion sessions themselves. Some of the issues around purpose and scope early in the project were handled this through 'time-out' of the intended agenda and additional individual and group conversations. In some instances, the core team were tasked with proposing a compromise outside these sessions and submitting this to the next session for approval. Given the positive working environment within the TFGs, in no instances was it not possible to agree a final version.

The main point of discomfort was at the start, particularly around the general understanding of purpose of the project and quality framework but secondly scope—whether it was on the quality of organisational/institutional approaches, rather than individual research projects.

This was handled through 'time-out' of the intended agenda and additional individual and group conversations about purpose and scope to come to a consensus.

## Results

### Outputs from the TFGs and piloting

**Fig. 2:**
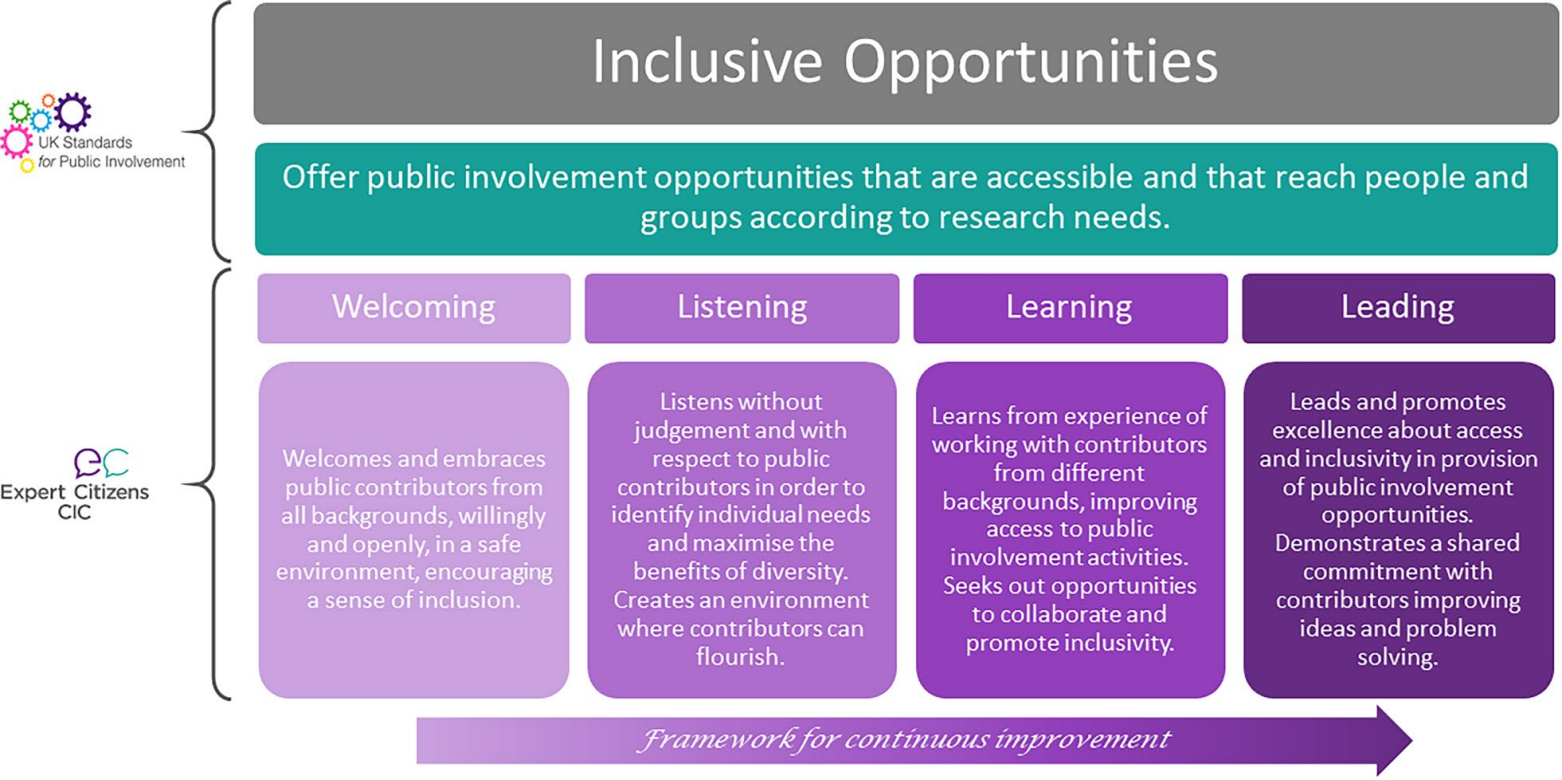
Quality Level descriptors the inclusive opportunities standard for the Insight | Public Involvement framework

Table 1 shows the composition, key aims, questions addressed and outputs from each of the four TFGs. The outputs are described in more detail in Additional file 3. An example of the co-developed Quality Level Descriptors is shown in Fig. 2, for the first of the UKSPI (*Inclusive Opportunities*). The changes made as a result of feedback from the piloting of the programme is described in Additional file 3.

### Reflections and learning

#### Online questionnaire

**Fig. 3:**
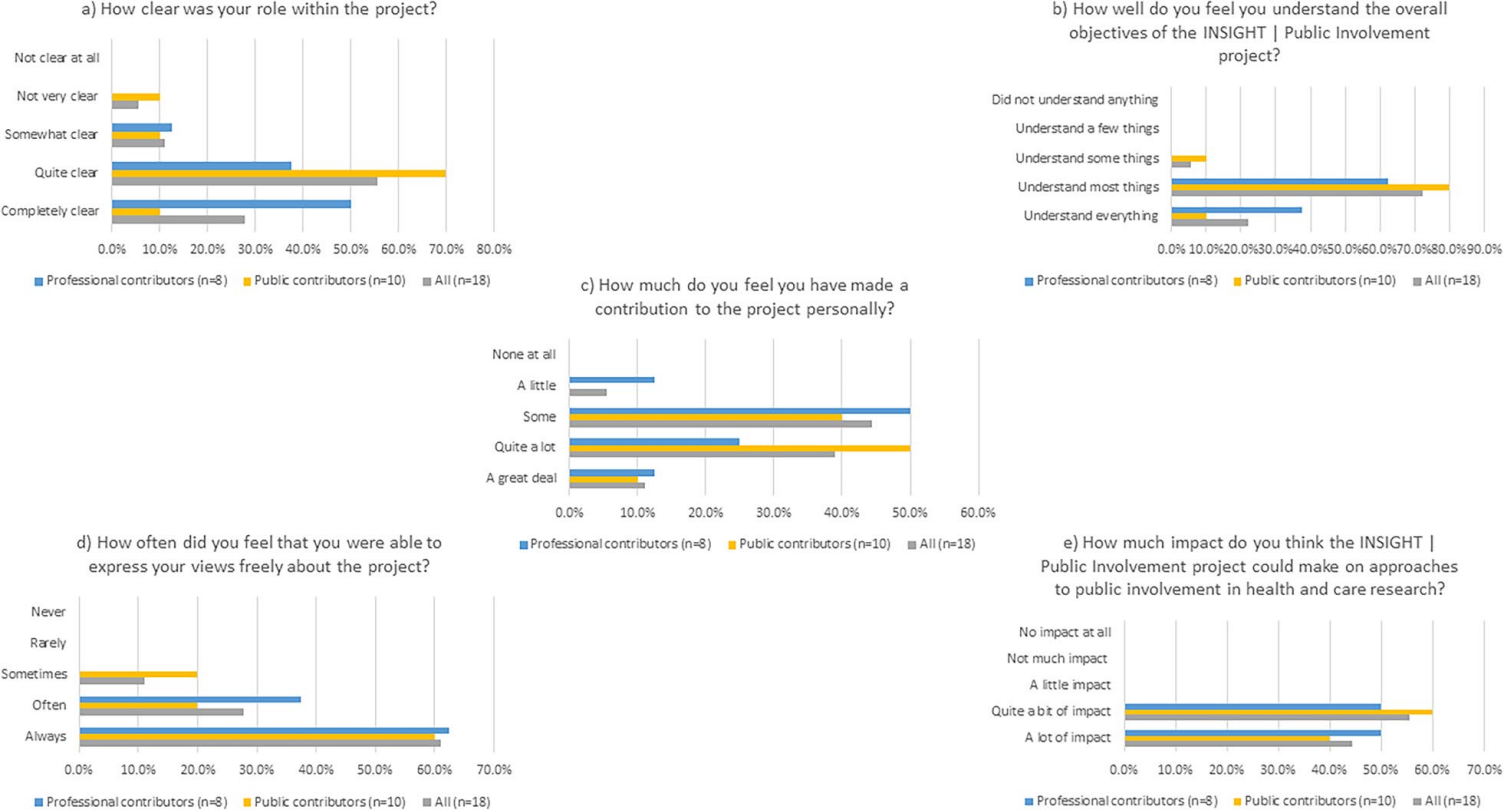
Views of project participants on their involvement in the project and its potential impact

The online questionnaire (Additional file 4) was completed by 18 participants in the project, comprising 10 public contributors involved in the TFGs (including 2 members of Expert Citizens), 4 staff from the three pilot sites, 2 steering group members and 2 project team members (Fig. 3). Overall, there appeared to be clarity regarding respondents’ role within the project and understanding of the project’s objectives with 15 (83.3%) being ‘*Completely clear’* or* ‘Quite clear’* of their role in the project, and 17 (94.4%) feeling that they understood ‘*Everything’* or ‘*Most things’* about the project’s objectives. Individual comments, particularly from those who were less clear about roles or objectives were used to inform future learning as the project proceeded. One public contributor felt that, ‘*Learning as the project progressed was welcome although I felt lack of knowledge at the beginning held me back.’*

All respondents bar one (94.4%) felt that they had made at least ‘*Some’* personal contribution to the project with half stating they had made ‘*Quite a lot*’ or ‘*A great deal’* of personal contribution*.* In addition to taking part in the TFGs, public contributors said that they were able to draw from their previous experience in both their previous professional experience or other PPI activities. One participant said; ‘*I have tried to give my thoughts on experiences of a past Quality Award Scheme from my previous profession and combine hopefully with new proactive ideas gained from many years of* [PPI] *experience in research.*’

In terms of freedom to express views during the project, 16/18 (88.9%) respondents said they were either ‘*Always’* or ‘*Often’* able to express their views freely about the project. Of the remaining two who stated that they were ‘*Sometimes’* able to express their views freely, one indicated that this improved as the project progressed. All respondents felt that the project would make either ‘*A lot of impact*’ or ‘*Quite a bit of impact*’ on approaches to public involvement in health and care research.

In general, results from public contributors were similar to those from professional respondents (Fig. 3).

In addition to the specific closed questions, the questionnaire provided open questions to provide opportunity for participants to reflect on their experiences. Table 2 provides examples of these reflections from the public contributors.

**Table 2:** Reflections from public contributors from the online survey

	Question	Quotes from public contributors
1	Please describe how, if at all, you have made a contribution to the project personally	*I have tried to give my thoughts on experiences of a past Quality Award Scheme from my previous profession and combine hopefully with new proactive ideas gained from many years of* [PPI]* experience in research* *As a member of the task and finish group, I felt I played a part in producing the training package using my 10 years* [PPI] *experience* *I used my lived experience to help lead conversations along with my knowledge of the* [Expert Citizens]* systems* *Being able to give views about what was discussed. And some of the things spoken about taken on board* *I would have liked to have been more involved from the start as initially I felt unprepared and lacking knowledge in the process* *I was able to bring my lived experience of mental issues and knowledge of the VCSE [Voluntary, Community and Social Enterprise] sector to the meetings I attended* *Giving context, explanations and examples in the task and finish group and also contributing to the wording*
2	What, if anything, have you learnt from your experience of being involved in the project?	*A much greater knowledge of the wider and excellent community work in* [PPI] *in our region and in different health care settings. To understand how best practise can be shared to improve all* [PPI]* standards* *How we can motivate and educate by recognising quality of good practise many great examples of community work in healthcare practise* *Good team working* *How different it needed to be for health and research* *Being part of a project that you helped with and seeing it from start to finish* *Learning as the project progressed was welcome although I felt lack of knowledge at the beginning held me back* *I have learnt that there is a wealth of human resources in our community—committed to and passionate about improvement and change* *How adaptable the standards are. Also learnt about the public involvement both at Keele and UHNM [University Hospitals of North Midlands] within different departments*
3	Please use this space to explain any of your answers further or provide any other comments about your experience of being involved with the project?	*This has been a steep but enlightening and rewarding learning curve for many working in different healthcare settings to firstly be made aware of the breadth of* [PPI] *work being carried out within the community then seeing how great examples of best practise can benefit everyone. At times the work has been challenging to consider ways and how we consider standards against our present working patterns but with great energy and fantastic ideas it is exciting to see a pilot Award scheme in its final stage. It’s been a great team effort and a great example of co- production* *If this can really take off, it would help with NIHR reviews if researchers belong to an organisation which has been evaluated* *Just being involved and seeing the project grow was so rewarding. Plus all the hard work of everyone involved. And how people were listened to* *I felt detached initially and would have liked more information regarding background to the project. I found the separate meeting with Expert Citizens provided me with a clearer understanding of the fundamental requirements* *Would like to see how this project works in practice and all the learning embedded in the next phase*

#### Review meeting

The review meeting at the end of the TFG sessions addressed 5 themes (Table 3). This provided very valuable learning, some of which was raised earlier in the project and was responded to as the project progressed. Overall, the response was positive for what we agreed was an ambitious project.

**Table 3:** Summary of key reflections from public contributors from the review session

	Review topic	Feedback from public contributors
1	The first steps: What were the initial challenges? What might we have done differently? What did we get right from the get go?	It was accepted by all that what we were trying to achieve as admirable and important. One public contributor said she ‘loved the idea’ It was felt that the initial idea was straightforward One contributor said that the project appeared too complex and questioned whether the remit was too wide Some felt ‘slightly overwhelmed’ by the size of the task One said ‘What a challenge!’ and wasn’t sure how it would go at first It was felt that we needed to introduce the contributors to the work of Expert Citizens and their model at the beginning It was agreed that the UKSPI were a good starting point rather than beginning with a blank canvas
2	The format and structure of the Task and Finish group meetings: What worked? What didn’t? How could we have improved it?	There was broad agreement that the TFG format worked well The overall size of the groups and proportion of public contributors was seen as appropriate One felt that the pace of the meetings was about right Early on in the process, we recognised the benefit of smaller sub-groups for specific topics and this was raised as a positive move in the review session
3	Contribution: What was your experience of participating in the Task and Finish groups? Did you feel you had opportunity to contribute? Did you feel that your views were listened to?	A number of contributors said that they felt listened to One said that there was ‘so much positivity’ in the meetings and that ‘all had something to offer’ One contributor stated that we ‘learn a lot from each other’ It was accepted by one participant as the development of the framework was an ‘iterative process’
4	Co-production: Co-production is about shared ownership, power and decisions as well as respect, openness and value. Where did we succeed in achieving this? Where did we miss this?	There was agreement among the public contributors that we had achieved our goal of co-production Several shared their previous experiences where co-production was claimed but not achieved One contributor said she was ‘amazed at how much had been done’ One member felt that the Core Team should have had more lay input
5	Communication: Did you feel that the communication was adequate? Appropriately worded?	It was generally felt that communication during the TFGs was appropriate One member said that she didn’t know much about it when the project started, highlighting the importance of adequate information before the start of the TFGs One contributor felt that updates following completion of each TFG could have been more frequent

#### Pilot site feedback

We also requested feedback from the pilot sites specifically on the format, content and potential impact of the final report. In terms of format, the pilot sites felt that the report looked ‘*professional’* and ‘*easy on the eye’*, but there were suggestions for minor changes such as page numbering, spacing and use of tables to summarise findings. The feedback was very positive regarding content with one site commenting that the ‘*suggestions for positive change section and the observations and evaluation sections for each standard were particularly helpful’.* It was felt that the report was too long and suggestions were made around revising this, which were adopted during the revision of the report template. Regarding potential benefits and impact of the report, pilot site participants felt that it informed the departments PPIE strategy and that participation in INSIGHT programme would ‘*help with grant applications and when recruiting public contributors*’.

#### Other reflections captured during the project

Several other themes were identified from feedback during the project, including: the general sense of the complexity and potential impact of the project, the feeling that contributor views were heard and shaped the final product, the importance of opportunities to address specific aspects in small sub-groups outside the TFG sessions, and the overall sense that the participants enjoyed the process.

These key themes were encapsulated during one of the TFG sessions (TFG 2, session 3) where, as one of the usual ice-breakers, the facilitator asked members to give one word to describe this project. Words used by participants included: ‘*Involvement’, ‘Share’, ‘Relieved’, ‘Challenging’, ‘Excited’, ‘Learning’, ‘Interesting’* and *‘Wow!’.* Despite being an informal tool to gauge the feelings of the group, it appeared to reflect very positive engagement in the project.

We also collated quotes from members of the first TFG when they were asked about their experience of participation at the end of the sessions. (Table 4).

**Table 4:** Reflections from members of Task and Finish groups

Quotes from participants
‘*Working with such a large and widely diverse group was a new experience for me. I feel the project benefitted from this, as we were all motivating each other, and plenty of ideas were "bounced" between us.*’ (Public contributor)
*‘I really like that everyone's thoughts, ideas and opinions are listened to and carefully considered. I think that type of environment is really key to having successful and productive sessions, which I feel we have had.’* (Clinical Trials Manager)
*‘They have really done a great job with the wording to keep it simple and meaningful. We can often get carried away putting too much in’* (PPI Manager)
*‘The process and wording we have followed has simplified a lay person's understanding, and as a lay person I can say that with confidence. Our understanding of the process is better as we have discussed and listened to everyone's views therefore a balanced and open view has been reached within the group.’* (Public contributor)
*'I am really enjoying being part of this project and being able to contribute to the work of the Task and Finish Group 1. There has been real evidence of co-production within this group. Everybody is listened to, their contributions valued, and everybody has worked well together. This I feel is reflected in the quality of the standards document that has been produced and the project as a whole will benefit from this’* (Head of PPI)

Further detail on the impact of public contribution and changes made as a result of feedback are highlighted in Additional files 1 and 3.

## Discussion

We believe that the Insight | Public Involvement Quality Recognition and Awards Programme represents the first co-produced programme that recognises, celebrates and shares how organisations and individuals involve the public in design and delivery of health and care research, using a strengths-based, appreciative inquiry approach. It is based on a collaboration between NHS, academia and community sectors, as well as strong public contributor representation. It also acted as an exemplar for the type of working together that the programme aimed to inspire in its participants.

### Other public involvement recognition schemes and quality frameworks

There are a large number of frameworks aimed at supporting, evaluating and improving the quality of public involvement though, in their review of these frameworks, Greenhalgh et al. [4] concluded that ‘*most published frameworks have been little used beyond the groups that developed them*’. They offer suggestions on how to develop a suitable framework and recommend working with ‘*patient collaborators*’ to ‘*plan and deliver a series of co‐design workshops to generate a locally relevant and locally owned framework*’. We broadly adopted this co-development approach with the intention of creating a programme that would have wider applicability and provide a mechanism for facilitating greater interaction between involvement practitioners.

We acknowledge that our programme is not the only initiative that offers awards for PPI activity. Over the last decade, regional NIHR Clinical Research Networks (CRN), including the West Midlands CRN, have had annual awards that recognises high quality PPI activity [17, 18] while the NIHR School for Primary Care have also offered prizes for public involvement [19]. These generally focus on specific PPI initiatives by small teams, rather than at organisational level. Our programme has a much wider reach and more systematic approach, with a drive to improve best practice, and facilitate its spread across healthcare and social research-active organisations.

The Engage Watermark is an award granted by the National Co-ordinating Centre for Public Engagement to higher education institutions to recognise their strategic support for, and commitment to improve, public engagement [20]. It is aimed at institution/faculty level, also has 4 levels (bronze, silver, gold, platinum) and provides a set of recommendations for improvement. Its reach extends beyond healthcare research but is limited to the higher education sector. It appears to use more of an audit and benchmarking approach and therefore feels more of an accreditation programme than the quality recognition approach that our use of appreciative inquiry brings. Importantly, its focus is on public engagement (attracting public interest in the research an organisation is doing) rather than involvement (including public contributors in the design and delivery of healthcare research). The Watermark programme therefore has some similarities to our programme and there may be mileage in drawing from this expertise.

Some universities have their own awards that recognise excellence in PPI activity [21, 22], though these are only open to the organisation’s staff members. At a project level, the European Research Council provide a *Public Engagement with Research Award* [23], designed to recognize and celebrate European Research Council grantees who have demonstrated excellence in public engagement and outreach. They offer monetary prizes (€10,000) for each of three awards—Involve (citizen science), Inspire (public outreach), Influence (media and policy). Winners are invited to a European Research Council-affiliated conference and are given increased media coverage. While this programme also provides some useful concepts that may be valuable in the future development of our programme, its project focus and restriction to grant awardees limits its current usefulness.

As well as quality awards schemes, several other PPI quality improvement tools exist. For example, the Guidance for Reporting Involvement of Patients and the Public (GRIPP) provides a framework for reporting study-level capture of PPI activities [36]. This is used extensively and extremely valuable for outlining PPI activities for projects and its embedding in the process for publication for journal such as *BMC Research Involvement and Engagement* encourages improvement in PPI quality.

The CUBE framework, was developed as a tool to help with planning and evaluating the process of PPI in research [40, 41]. It examines the quality of PPI across four domains; the strength of the public voice, diversity in ways for public contributors to be involved, the degree of attention to public concerns and organisational attitude to change. Hence, there is overlap across some of the UKSPI standards and the workshop approach suggested CUBE would be a useful way to review and evidence the quality of PPI activity within an organisation as part of an INSIGHT self-assessment. In terms of the UKPSI ‘impact’ standard, the Public Involvement Impact Assessment Framework (PiiAF) represents an excellent tool to record and is ‘*primarily aimed at researchers who wish to design an assessment of the impact of public involvement in their research’* [41–43]. We propose that this would be a valuable tool to evaluate the effectiveness of INSIGHT as well as providing a means for collecting impact evidence for organisations who wish to participate in the INSIGHT programme. More recently, the Public Involvement in Research Impact Toolkit (PIRIT) has been co-developed by public contributors and staff members for use at the Marie Curie Research Centre and the Wales Cancer Research Centre [44]. It comprises tools to support researchers, particularly in public involvement planning and impact reporting at a project level. It aligns to the UKSPI and provides an excellent checklist to ensure public contributors are involved at every stage of a project, whilst also capturing their contribution and impact on how the project was developed and delivered.

While these schemes provide significant complementarity to INSIGHT and would represent means to collect organisational evidence of high-quality PPI for submission to INSIGHT, most are not aligned directly to the UKSPI and do not cover the breadth of PPI activity encompassed by the UKSPI. Furthermore, they do not celebrate or facilitate spread of PPI excellence, and are more focused on the benefits to an individual organisation or research team.

### Programme structure

As mentioned in the Introduction, the UKSPI shied away from the introduction of a ‘rating’ system alongside their six standards. We too wanted to avoid anything that hinted at an accreditation scheme or audit process as this was felt to be a disincentive for participants. However, the use of quality levels in the context of the Expert Citizens Insight Evaluation programme [25], underpinned with an appreciative inquiry, strengths-based approach, was felt to be a workable model as a positive means of identifying existing good practice and support continuous improvement. Our public contributors embraced this approach, and the pilot sites appreciated the support in facilitating ongoing improvement that this approach gave them. The ability to quote the achieved level in grant applications and on organisational marketing material was felt to be a positive incentivisation for participation by the public contributors and pilot sites.

### Benefits afforded by the INSIGHT programme

As summarised above, we believe that our programme brings together key elements of existing initiatives to create a programme that has several important, often unique, benefits:

#### Flexibility

Given the generic approach we have used, it was felt that INSIGHT could be suitable for national or even international application. By incorporating within the assessment process a mechanism to account for organisation size and resources it is flexible enough to be applicable to any type of organisation (i.e. any involved in health and social care research; including commercial research organisations such as pharmaceutical companies, contract research organisations and medical equipment manufacturers, as well as those small third sector groups or charities that are frequently overlooked). Furthermore, by combining both the INSIGHT assessment scheme for organisations and departments alongside the INSIGHT quality awards event for individuals and small teams, it accommodates any PPI-active groupings from individuals to whole organisations. This would address some of the limitations of existing programmes as described above [17–23].

#### Independent, lived experience-led assessment

It provides an independent assessment of public involvement activity, co-led by public contributors, which would assist in validating statements made on grant applications, websites, etc., regarding public involvement activity within an organisation. While healthcare research funders and governance bodies such as research ethics and the UK Health Research Authority increasingly require evidence of public involvement at every stage of the grant development and delivery [10–13], independent assessment of this is sometimes challenging [9].

#### Profile

The celebratory nature of the national annual Awards Event provides the opportunity to raise the profile of public involvement activity. It also provides opportunity to demonstrate the importance and celebrate the impact of PPI activities. While some current public involvement activities are publicised, their reach is often more locality-/region-focused [17, 18, 21, 22], or project-based [23].

#### Spread and Improvement

The Awards Event (particularly the use of a special award for spreading improvement), along with our plans for a repository of involvement ideas, training and toolkits, facilitate the spread of public involvement activities across health and social care research organisations. Such a repository could supplement and align with the NIHR CED’s Learning for Involvement website [45]. We are not aware of such a central, national resource of approaches and ideas for improving PPI, though it is believed that there are plans for the NIHR to bring all their PPI information into a single resource centre (CED—personal communication). Furthermore, the programme aims to expand the number of public contributors; directly by encouraging new public assessors to be part of the assessment process, and indirectly by encouraging participating commercial and non-commercial organisations to improve their PPI activity. It also supports the drive for improvement in the level and quality of public involvement at individual to organisational level and creates an incentive to develop strategic approaches to this activity. While other organisations, including the NIHR CED and CRN PPI networks [14, 17, 18], exist to facilitate spread and improvement in public involvement activity, our aim is to work with these groups to expand this further using a systematic approach based on a common set of quality indicators and levels.

#### Incentivisation

We feel that our focus on a ‘carrot’ (pull) rather than ‘stick’ (push) approach to improving public involvement, by use of an appreciative inquiry approach with celebration of best practice rather than an audit-based programme, is more likely to incentivise participation in the scheme. While programmes such as the Engage Watermark [20] has significant potential for expansion from engagement into involvement, and across to healthcare organisations, we felt that its audit style approach may disincentivise participation and may go against the principles behind why the UKSPI were developed [15].

#### Innovation

Our programme encourages innovation in public involvement activities and this is further recognised by the special award for innovation as part of our Quality Awards Event. In the context of the ever-changing technological landscape and use of social media, not to forget the innovations brought about by the COVID-19 pandemic, our aim was to encourage new ways of expanding public involvement.

#### Equality, diversity and inclusion

Equality, diversity and inclusion represents a key priority for the NIHR [46], including in public involvement activity [47]. Our programme aims to address the general lack of diversity in public contributors, including by encouraging equality, diversity and inclusion in membership of assessment panels and by the use of a special Encouraging Diversity Award.

#### Impact

Our overall aim is to improve the quality and relevance of health and social care research and we firmly believe that greater public involvement is a key component of this. Numerous studies have indicated the potential ways in which public involvement may improve clinical research, though how these can be accurately evaluated remains a topic of debate [12, 48]. Public involvement is not only about impact on the research itself, but may also encompass benefits such as empowerment of public contributors and improving the wider relationship between the public and researchers [4], as well as making health and social care research more accessible to the public [6]. Some have suggested that the potential negative impact of public involvement has been little studied [48], though while such balancing measures should indeed be included in evaluation of impact, it is difficult to imagine that harms would outweigh the benefits. For example, our public contributors have described how their health literacy improved through involvement in PPI activities.

### Co-production

As noted above, Greenhalgh et al. [4] identified 65 frameworks related to co-production in research. These frameworks generally focused on their use within individual projects and targeted specific aspects such as power-sharing, participant recruitment, research priority-setting, report writing or partnership development.

While we did not adopt a specific framework, we aimed to incorporate the key principles of co-production covered by these frameworks in the development of our programme, together with an equal power partnership in its delivery, in order to ensure that the public voice is genuinely represented in a scheme focusing on such a topic. It became clear during the task and finish groups that public contributors ask questions and generate ideas that would not readily be apparent to academics. Wicks et al. [11] stated that ‘*One of the main stumbling blocks to “coproduction” of research with patients and the public is that professionals lack knowledge, skills, and experience on how best to do it*.’ While the NIHR have published guidelines on co-production [35], our experience from this study illustrated that clinical and academic interpretations of this term vary considerably and that true co-production is rare. Indeed, one benefit from involving Expert Citizens as a core partner was that they focus heavily on co-production and have delivered workshops on the subject from the point of view of people with lived experience [49], thereby reversing the usual balance of ‘professional teaching the public’ in this regard.

In terms of the project itself, we brought together ‘professionals’ with public contributors from very different backgrounds in the TFGs. Given that co-production, with its bringing together of people with different and frequently strongly held views, can itself be challenging at times [24, 35], we expected some challenging TFG sessions I this regard. However, while the breadth of expertise and experience inevitably generated a diverse range of views, consensus was overall very easy to reach and we did not encounter any instances on overt or intractable disagreement.

### Reflection and learning

In addition to gaining feedback on the materials we developed throughout the project, and making revisions accordingly, it was important to capture the learning and reflections from public and professional participants. On the whole, comments from public contributors suggested a very positive experience and enthusiasm for the project. There was an overall sense of genuine co-production with the freedom to express views and see those views initiate change. However, we learned that it was important to clarify roles and take time to explain the background and aims of the project at the beginning, particularly as it was recognised that these aims were ambitious. Similar to previous observations [50], adapting to the needs of individual public contributors was important and we identified that smaller group sessions outside the main TFGs was extremely valuable for some participants. In a systematic review of patient engagement in research, Domecq et al. [1] also identified that spending adequate time to build reciprocal relationships between public contributors and researchers, fostering mutual respect and being clear on what is expected of public contributors were seen as important.

Our initial plan was for the TFGs to operate as face-to-face meetings. However, the COVID-19 pandemic-associated restrictions prevented that and all groups were conducted using an online platform. This was disappointing in respect to the reduced ability to generate the inter-personal interactions and bonding between group members. However, notwithstanding a few technical glitches at times, all participants adapted to the online format, and we were able to hold a larger number of sessions due to reduced costs. As also experienced by the Blueprint Writing Collective [50], a hybrid approach has become a more common feature in the Impact Accelerator Unit at Keele University (the Unit which leads on PPI within the University’s Medical School), though we are mindful of the risks of digital exclusion.

We also identified that working alongside a third sector organisation comprising people with lived experience (Expert Citizens) and who had created the original Insight framework, provided a bridge between the health-associated professionals/academics, and those public contributors who were unfamiliar with the Insight concept. This use of third sector organisations to facilitate meaningful public involvement is not new [50] and can facilitate building bridges that otherwise might be challenging to academics working independently.

The professional members of the team also recognised their own learning from interacting with an array of public contributors with diverse experiences and expertise. In our project, the academic team aimed to use a co-production approach from the start. However, it can be challenging to adhere to *‘… principles of respect, trust, reciprocity, and co-learning…*’ (as described by Kirwan et al.as one of their core guidelines for patient engagement [51]) as these take significant time and commitment to adopt by researchers who are used to driving the research agenda themselves. During the project, the immense value of the experience and expertise provided by public contributors became very clear, making these guidelines much easier to follow. The power of the public voice may have been assisted by the fact that we elected to involve a large number of public contributors (almost half of TFG members).

### Challenges and next steps

We recognise that this programme is in its infancy and requires significant further evaluation and refinement, as well as continuing support from key stakeholders such as the NIHR, before it becomes a fully-fledged programme. To this end, we have been encouraged by the CRN West Midlands’ support for the project to date and by their continued commitment to support a regional roll-out and evaluation of the Insight | Public Involvement programme across NHS partners within the region. The evaluation will present challenges, as illustrated by Russell et al. [48] and Boivin et al. [12], and will need to include an appropriate a mix of outcome, process and balancing measures using a mixed methods approach. This, along with a realist evaluation INSIGHT to determine how it could be adapted for different health and social care settings, forms part of the next steps of the programme’s development.

We also acknowledge that a sustainable business model needed to be developed, along with the associated marketing strategy and allied commercial considerations. These aspects formed part of phase 2 of the programme’s development, which included independent market research and collaboration with the Chamber of Commerce to develop the business model. This indicates that the programme is sustainably over the long term.

While the programme was developed so that it can be applied across most settings where health and social care research is carried out, including the private sector, the pilot work has focused on the UK NHS and academia. It may require further refinement to be applicable more widely (e.g. commercial sector, social care, third sector organisations) and beyond the UK. However, we believe that the core structure is sufficiently flexible and resilient so as to not require wholesale changes.

## Conclusions

We have co-produced the framework for a Quality Recognition and Awards Programme that celebrates and facilitates the spread of public involvement in health and social care research. We are not aware of a similar programme that fulfils this need. As the field of public involvement in health and social care research continues to grow, we believe that this programme will facilitate this growth, provide a core repository of involvement ideas and indeed potentially act as a mechanism for evaluating its quality and impact. We also believe this framework promotes the value and benefit to the public contributors, with the potential for increasing their numbers, time and commitment.

### Supplementary Information

**Additional file 1.** GRIPP2 short form.

**Additional file 2.** The Insight | Public Involvement programme: concept, project oversight, project launch event and co-production approach.

**Additional file 3.** Programme development outputs and impact of piloting. **Figure AF3-1** GANTT chart showing the timings of some key activities. **Figure AF3-2** Components of the Insight | Public Involvement Quality Recognition and Awards Programme. **Figure AF3-3** Overall structure of the Insight | Public Involvement Quality Recognition and Awards Programme. **Figure AF3-4** Process maps for the (a) Quality Recognition Scheme and (b) Quality Awards Event

**Additional file 4.** Bespoke online feedback questionnaire.

## Data Availability

All data generated or analysed during this study are included in this published article.

## Abbreviations

PPI: Public and Patient Involvement
UKSPI: UK Standards for Public Involvement
NIHR: National Institute for Health Research
CED: Centre for Engagement and Dissemination
TFG: Task and Finish Group
CRN: Clinical Research Network
