# Supplementary material for: The INSIGHT project: reflections on the co-production of a quality recognition programme to showcase excellence in public involvement in health and care research
Source: Res Involv Engagem. 2023 Oct 25;9:99. doi: 10.1186/s40900-023-00508-4 (PMC10601214; doi:10.1186/s40900-023-00508-4)
Supplement: Supplementary file 1 — Additional file 1. GRIPP2 short form. [file 40900_2023_508_MOESM1_ESM.docx]

**Additional File 1 GRIPP2 Short Form**

| Section and topic | Item | Reported on page no. |
| --- | --- | --- |
| 1: Aims | To utilise the UK Standards and Expert Citizens Insight Evaluation Programme to:   - Co-produce a quality recognition and awards programme with public contributors that recognises, celebrates and shares excellence in public involvement across the full spectrum of health and social care research settings. - Capture and reflect upon the way in which co-production involving public contributors at all stages influenced the development of the programme, thereby illustrating the power of this approach. | End of Background section |
| 2: Methods | Partnership with Expert Citizens C.I.C. (EC; led by those with lived experience of multiple disadvantage) allowed access to, and adaptation of, their Insight Evaluation Programme. EC worked in partnership with the academic team to develop the funding application and contributed to the presentation to the funding panel.  EC hosted the Launch Event, at which public contributors comprised 51% of the stakeholders and inputted into the scoping of the project.  Public contributors comprised 41% of the Task & Finish Groups (TFGs). Principles of co-production, with emphasis on equality of power, were outlined at the beginning of each TFG. As equal partner in the TFGs, they co-created the programme framework, developed quality indicators, co-produced the assessment framework, co-created the assessor training package and co-developed the format and logistics for the Quality Awards Event.  Separate public contributors reviewed the assessor training package.  They acted as assessors for the pilot site assessments, and provided feedback on this experience  They were also involved as contributors to the pilot site assessments as assessees by participating in individual conversations with the assessment panels (as part of the programme assessment structure). Public contributors provided reflective feedback on the development of the programme, both as responses to a questionnaire and during review meetings. | Methods section  Table 1  Figure 1-2  Additional files 2 & 3 |
| 3: Study results | Public contributors added value to the programme in numerous ways:   - EC members provided considerable expertise and experience on co-creation and delivery of their Insight Evaluation programme. - EC members were also key to co-writing the funding bid by outlining their Insight model, providing costs and attending the funding panel presentation (including creating slides and speaking at the event). - EC members hosted the Launch Event, utilising their experience of breakout rooms in Zoom. - At the Launch event, public contributors helped to scope out the broad framework for the programme, including recommending that existing frameworks were utilised (UK Standards and EC Insight models). They also raised important design considerations that fed into the aims of the TFGs, future considerations regarding scaling up and endorsement, and conduct of the pilots. - As members of the TFGs, public contributors made significant impact on the programme framework structure, co-created the wording of the quality indicators, co-designed the assessment framework (including creation of the process maps for the programmes two main components, development of the self-assessment forms and other outputs [see Table 1]), co-created an assessor training pack, and developed the framework and format for the Quality Awards event (including creating the nomination form and adapting the training package. - During the pilots, public contributors acted as assessors and provided feedback on the process, which lead to revisions of the documentation. - At the conclusion of the project, public contributors completed a questionnaire on their experience of the co-production process and participated in a review meeting to explore their experience of taking part. They were members of a writing group that helped create the framework for this article and review its content. As such three members are listed as co-authors. | Results section  Additional files 2 & 3  Table 1-4  Figure 3 |
| 4: Discussion and conclusions | Overall, public contributors had a major impact on shaping both the overall design and detail of the programme and its outputs. This included aspects such as the importance of moving away from a language of an 'evaluation' or 'accreditation' programme to one of incentivising and celebrating best practice using an appreciative inquiry approach. They redesigned the format of the TFGs to include separate small groups to address individual issues. Several members had prior experience of working on local, regional and national groups as PPIE representatives and brought this experience to the discussions.  Expert Citizens members provided unique contributions as they comprised a group often under-represented in healthcare research. Their experience of delivering their Insight Evaluation programme was invaluable and other public contributors as well as the academic team recognised the critical insight that their experience and expertise brought to the project.  Even with this wealth of experience, we recognised that there were still groups that were still under-represented in our public contributor team. We lacked the diversity of cultural backgrounds that we would have liked and have specifically identified an Equality, Diversity and Inclusion review as an important next step.  Due to the timing of the project during the pandemic, we needed to adapt the TFGs to an online format. We wondered how this would work initially, but public contributors soon adjusted to the format, despite a few technical teething troubles. | Discussion section |
| 5: Reflections/critical perspective | Our aim from the beginning was to follow a co-production approach as closely as possible. Feedback from the public contributors suggested that they felt that they were able to express their views freely and that they felt they had made a significant contribution to the project. Some public contributors felt that they initially did not understand the aims of the project, especially if they were not involved in the first TFG. This highlighted the need to make sure that contributors were provided with sufficient background information at the beginning of their involvement, particularly given the complexity of the project. | Results section  Tables 1-4  Figure 3 |
