# Supplementary material for: The INSIGHT project: reflections on the co-production of a quality recognition programme to showcase excellence in public involvement in health and care research
Source: Res Involv Engagem. 2023 Oct 25;9:99. doi: 10.1186/s40900-023-00508-4 (PMC10601214; doi:10.1186/s40900-023-00508-4)
Supplement: Supplementary file 2 — Additional file 2. The Insight | Public Involvement programme: concept, project oversight, project launch event and co-production approach. [file 40900_2023_508_MOESM2_ESM.docx]

**Additional file 2 The Insight | Public Involvement programme: concept, project oversight, project launch event and co-production approach.**

Initial concept

During discussions between staff from Keele University’s Impact Accelerator Unit (AAF, SB), and the Expert Citizens team (DM, AM, RH), the new framework was conceived as a hybrid programme that brought together the model used in the Expert Citizens Insight Evaluation Programme^[[1]](#footnote-1)^and the UKSPI^[[2]](#footnote-2)^.

The Expert Citizens Insight Evaluation model utilised an appreciative inquiry approach to assess extent to which a department of organisation meets their Insight Standards. Each standard has four possible levels; *Welcoming, Listening, Learning, Leading*. These levels, while progressive in nature, all provide a positive description of the level achieved.

Project oversight

The project governance framework, each of which included lay input, consisted of:

- A short weekly Core Group meeting, comprising a small team of around four members (AAF, SB, RH, NE, JT) who were central to the day-to-day running of the project. This group maintained momentum and dealt with the day-to-day issues, reviewed progress and planned immediate next steps. Importantly, this included key input from Expert Citizens (RH) who provided lay input as well as experience and expertise from their Insight Evaluation Programme.
- A monthly Project Operational Group, chaired by the Project Lead (AAF). This comprised a broad group of individuals involved in the operational running of the project (including SB, AAF, DM, RH, SF, PP, NE, KL, JL) with a view to identifying and addressing potential barriers and general operational issues. As the project progressed, it became evident that this group was meeting too frequently to be an appropriate use of member time, and was therefore moved to bimonthly.
- A quarterly Project Steering Group, chaired by the head of the Keele University Impact Accelerator Unit (which coordinates PPIE activity within the University’s School of Medicine), but who was not directly involved in the project. It comprised project team members, non-involved representatives from the four organisations, representation from the funder, and lay input from both EC and the Keele University Research User Group. Their remit was: (i) to provide overall review of quality and to ensure that the project was conducted and delivered as described in the funded project application, (ii) to monitor and review progress of the project against milestones and budget, (iii) to provide advice to the project lead and management team, (iv) to receive Steering Committee reports on the project prior to each Steering Group Meeting and review and offer feedback, prior to reports being submitted to the funder, (v) to agree appropriate changes to the project plan as proposed by the project team and, (vi) to advise the project lead on dissemination and the presentation of all aspects of the project.

Launch event

At the start of the project, a Launch Event was held, hosted by Expert Citizens (DM, RH) and chaired by AAF. In total, 39 delegates attended. These comprised 20 public contributors (including 4 from Expert Citizens), representatives of the four organisations involved in the project and representatives from the Clinical Research Network West Midlands and the National Institute for Health Research Centre for Engagement and Dissemination.

The event comprised a welcome and introduction to the project by AAF, followed by two breakout sessions (using breakout rooms in Zoom hosted by DM and co-ordinated by RH). The breakout sessions comprised three separate groups of 12-14 delegates (with a chair, scribe and 4-7 public contributors), each addressing a separate topic. After each session, the groups came together to report on their discussions. Using a set of broad questions as a guide, the topics covered by the breakouts included the separate elements of the project:

- Session 1, group 1: Co-development of the quality indicators
- Session 1, group 2: The Assessment Framework
- Session 1, group 3: The Assessor Training Framework
- Session 2, group 1: The annual Insight Awards Event
- Session 2, group 2: Piloting the programme
- Session 2, group 3: Scaling and Endorsement

After the event, notes were collated by the core team (AAF, SB, RH, NE, JT) and utilised to provide the basis for the programme of work for the four task and finish groups (TFGs), the approach to the pilot phase and to inform the longer-term approach to expanding the project.

Outputs from the Launch meeting informed:

1. the key topics used at the beginning of the four TFGs
2. the selection of the pilot sites
3. the potential future challenges for scaling up and endorsement of the programme

During the Launch event, it was agreed that the UKSPI and Expert Citizens Insight Evaluation be used as a starting point, rather than starting with a blank piece of paper.

Task and finish group format

TFG members were sent a pre-meeting pack by the group chair prior to each meeting to provide background information on things to think about prior to the meeting, along with the meeting agenda. The structure of a typical TFG agenda included:

- a welcome and introductions (involving an ice-breaker to allow members to get to know each other better)
- outlining of ground rules (for the first meeting)
- review of the previous meeting (including opportunity for reflections from members and review of actions)
- a guided discussion on one or two key topics
- a summing up of the session with proposed actions.

Each meeting was ~90 minutes in length and chaired by AAF or SB. Notes were taken by a nominated scribe at each meeting to capture actions, reflections and learning.

1. Expert Citizens Insight Evaluation. <https://expertcitizens.org.uk/insightevaluation/> [↑](#footnote-ref-1)
2. National Institute for Health Research. UK Standards for Public Involvement. Nov 2019 <https://sites.google.com/nihr.ac.uk/pi-standards/home> [↑](#footnote-ref-2)
