# Supplementary material for: The INSIGHT project: reflections on the co-production of a quality recognition programme to showcase excellence in public involvement in health and care research
Source: Res Involv Engagem. 2023 Oct 25;9:99. doi: 10.1186/s40900-023-00508-4 (PMC10601214; doi:10.1186/s40900-023-00508-4)
Supplement: Supplementary file 3 — Additional file 3. Programme development outputs and impact of piloting. Figure AF3-1 GANTT chart showing the timings of some key activities. Figure AF3-2 Components of the Insight | Public Involvement Quality Recognition and Awards Programme. Figure AF3-3 Overall structure of the Insight | Public Involvement Quality Recognition and Awards Programme. Figure AF3-4 Process maps for the (a) Quality Recognition Scheme and (b) Quality Awards Event [file 40900_2023_508_MOESM3_ESM.docx]

**Additional File 3 Programme development outputs & impact of piloting**

**The outputs from the Task and Finish groups**

The format of the Task and Finish groups (TFGs) is described in Additional File 2. There were 21 TFG sessions (including a joint one between TFGs 2 and 3) over 33 weeks (Figure AF3-1), with a final combined TFG feedback session. Public contributors comprised 43% (19/44) of participants (a small number of public contributors took part in more than one TFG).

TFG 1 firstly defined the overall structure for the Quality Recognition and Awards Programme. Figure AF3-2 show that the overall programme comprised two elements: the Quality Recognition Scheme aimed at assessing public involvement within organisations or departments, and the Quality Awards Event; an annual awards event focusing on celebrating and sharing excellence in PPIE aimed more at individuals and small teams.

The TFG then created the quality indicators, using the six UKSPI*: Inclusive Opportunities, Working Together, Support and Learning, Communications, Impact, Governance*. Each of the six indicators had four incremental levels, based on the Expert Citizens Insight Evaluation Programme levels (*Welcoming, Listening, Learning,* and *Leading*) to create a matrix as illustrated in Figure AF3-3.

TFG 2 utilised the Quality Level Descriptors from TFG 1 and devised the assessment framework for the Quality Recognition Scheme component of the programme. The elements of this are summarised in a co-developed process map (Figure AF3-4a).

Consistent with our appreciative inquiry approach, the assessment was created to be iterative in nature, with opportunity for two-way conversation and requests further information as part of the self-assessment process. We also included individual conversations with public contributors as part of the assessment process to provide opportunity to allow public contributors to describe their experience. While a flexible approach to these conversations was preferable, a topic guide was created to provide some structure if required. We also wanted to gain some element of an independent view of public involvement activities and hence included individual conversations with someone from a partner organisation, such as with the Clinical Research Network PPIE lead.

The group also co-created an introductory pack and associated slide deck for participating departments/organisations to assist in describing the programme to those unfamiliar with its structure. Finally, the group adapted the report template used by Expert Citizens to create a template final report that would be completed by the Insight team for participating organisations.

TFG 3 examined the training requirements for assessors. Given the close links between group 2 and group 3 activities, we also held a joint meeting between these groups (see Figure AF3-1) to ensure consistence of approach to assessment. Early in discussions, it was decided that an assessment panel would comprise an equal balance of public and professional assessors each and that their training requirements would be the same. To this end, assessor role descriptors were developed.

A key part of group 3’s objectives was to develop a training pack for assessors. This incorporated training in the structure of the programme, the quality indicators and how to use them, the involvement of the assessors at each stage, assessor expectations, and mentorship. The group identified additional generic training required, such as report writing/reviewing, record keeping, communication skills and governance elements. The group also discussed recruitment of assessors, including the importance of equality, diversity and inclusion.

TFG 4 focused on the Quality Awards Event and addressed two key areas: (i) the nomination process and (ii) the logistics of the event. The group added content to both the introductory information and the assessor training pack, and created a process map specific for the Quality Awards event (Figure AF3-4b) and a short nomination form. The group felt that nominations could come from any source, including: staff within an organisation conducting health & care research, members of the public, by self-nomination, or the Insight | Public Involvement team (e.g. on the basis of the Quality Recognition Scheme case studies). It was agreed that nominators could opt to remain anonymous and that there would be no cap on the number of nominations from a given organisation.

Initially, 4 awards were identified, one for each of the four levels (*Welcoming, Listening, Learning, Leading*) and an adjudication process which included the same mix of public and professional adjudicators as used for the organisational-level assessment, with the option of a guest judge after an initial shortlisting stage. A winner and two runners-up would be selected for each award, to be presented a national annual awards event.

Logistically, the Quality Awards Event was adapted from the Expert Citizens National Insight Awards event^[[1]](#footnote-1)^, with guest speakers drawn from national leading figures in the field and previous Quality Recognition Scheme or Awards Event winners. It was proposed that this would provide a forum for network and sharing of ideas to facilitate spread of public involvement activity. The group also discussed funding/sponsorship, venue type and location, timing, content (e.g. running order, parallel sessions, workshops/breakout groups, networking), options for a poster competition, marketing, use of social media (for both marketing and in-event feedback) and branding.

**Impact of piloting on project outputs**

In response to feedback from pilot sites, several documents and forms were amended, including the introductory pack and slide deck, training pack, self-assessment form and final report template. Piloting of the training pack with new public assessors also led to amendments to the training pack and highlighted the importance of expanding the section on assessor expectations, remuneration and time commitments for assessors. We also amended the nomination form and expanded the number of awards from 4 to 7, to include special awards in three new categories (*Encouraging Diversity, Spreading Excellence, and Innovation*).

**Figure AF3-1 GANTT chart showing the timings of some key activities.**

**Figure AF3-2 Components of the Insight | Public Involvement Quality Recognition and Awards Programme**

**
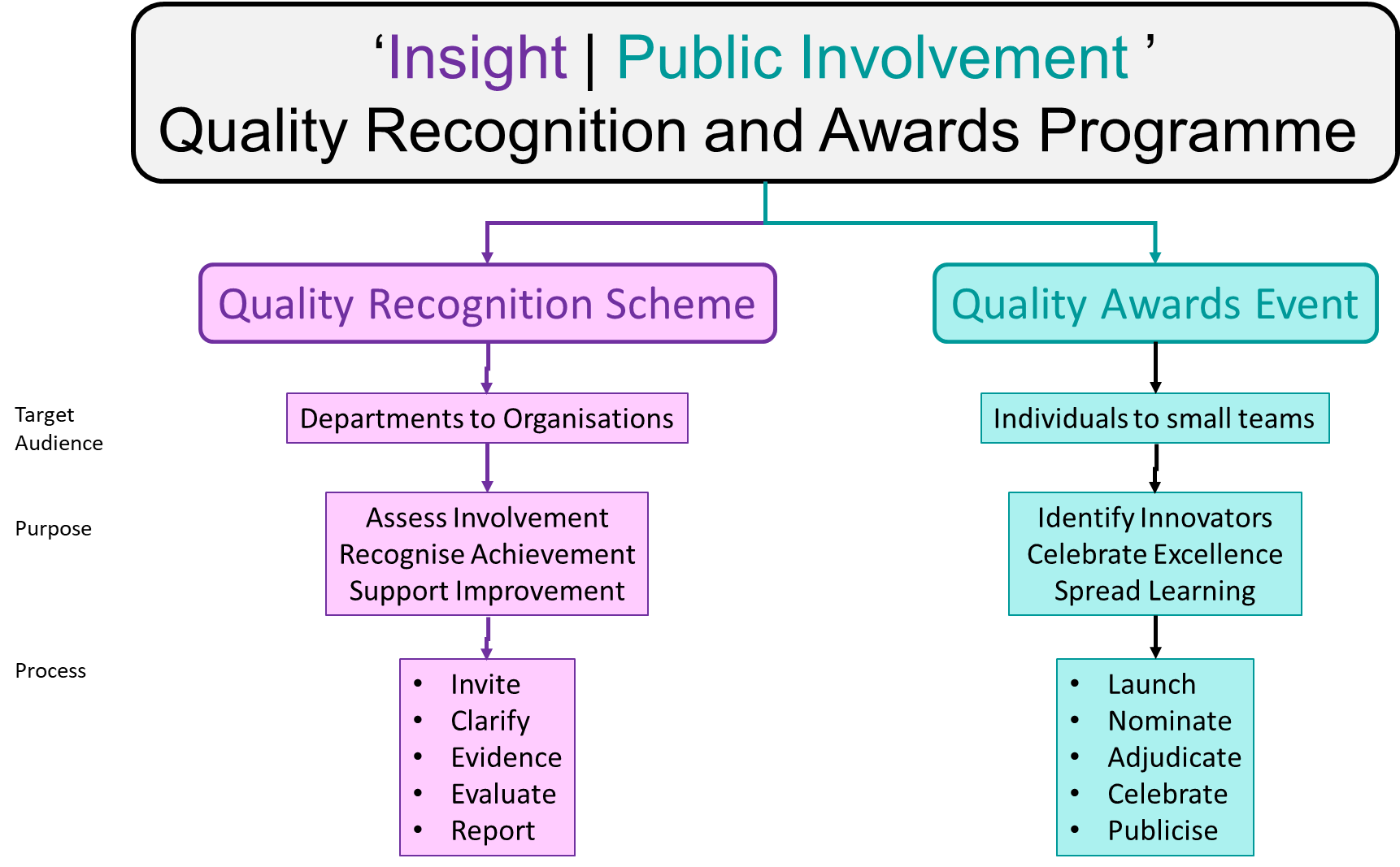
**

**Figure AF3-3 Overall structure of the Insight | Public Involvement Quality Recognition and Awards Programme**

**
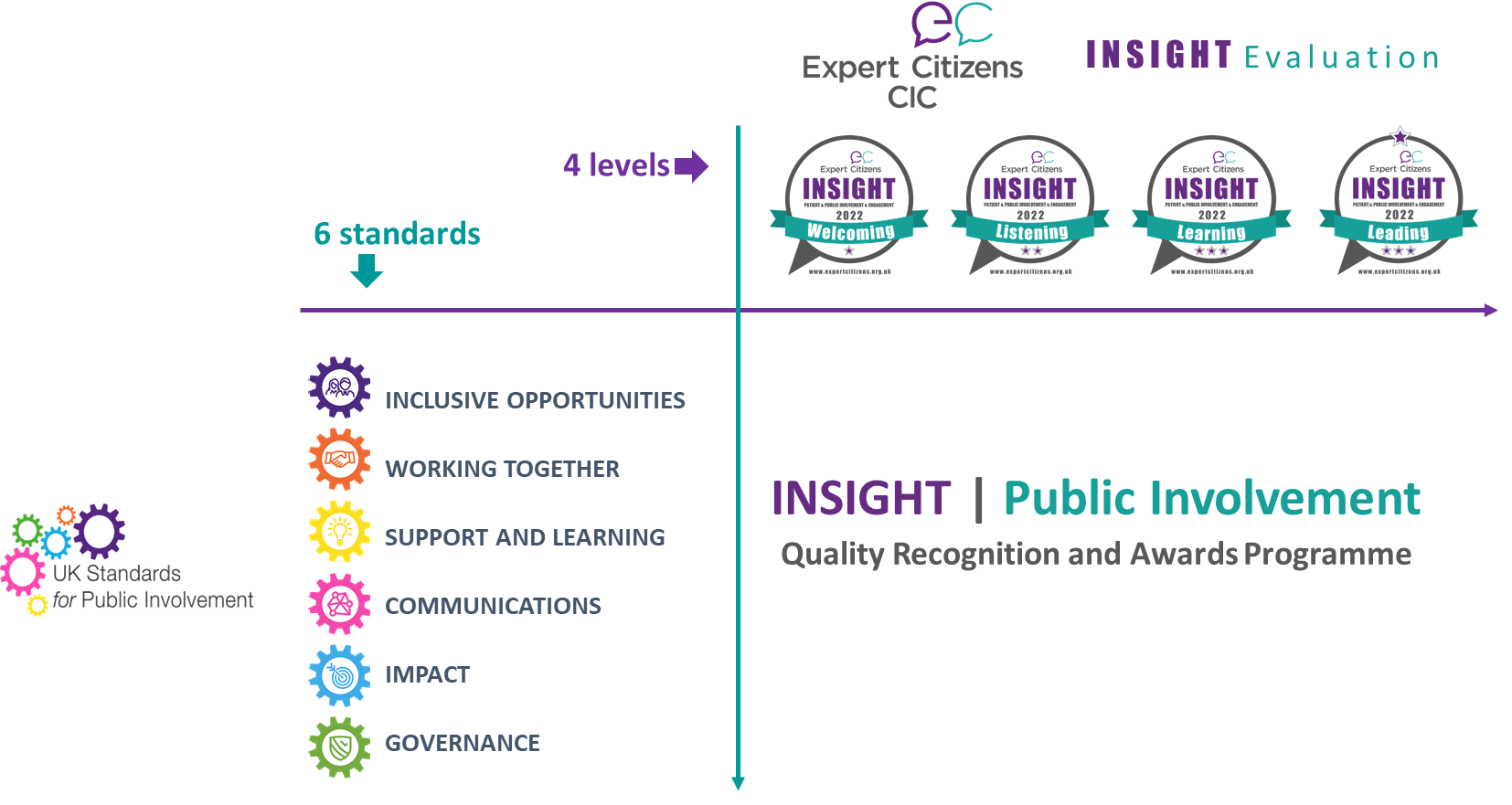
**

**Figure AF3-4 Process maps for the (a) Quality Recognition Scheme and (b) Quality Awards Event**

(a)

**
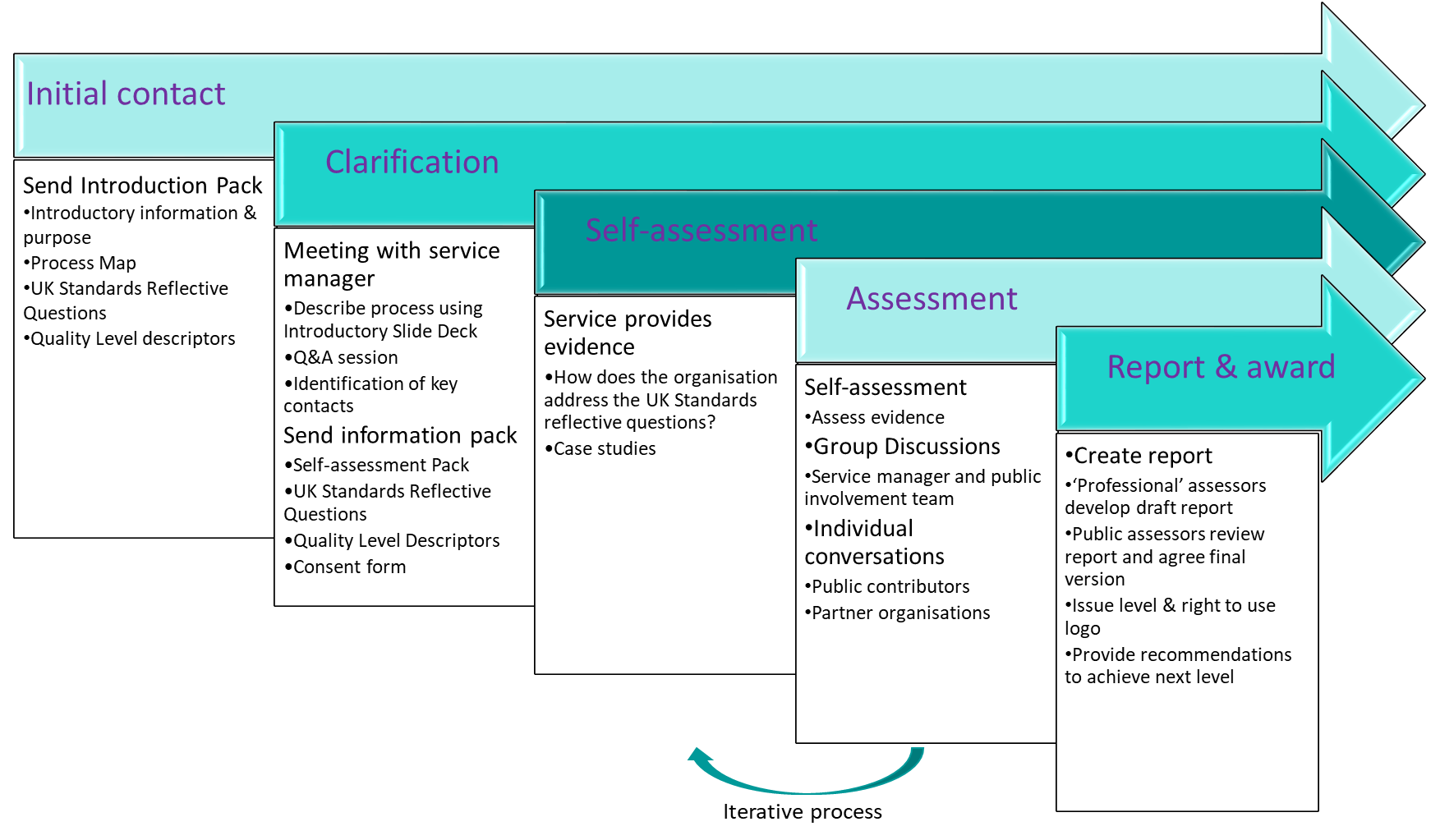
**

(b)


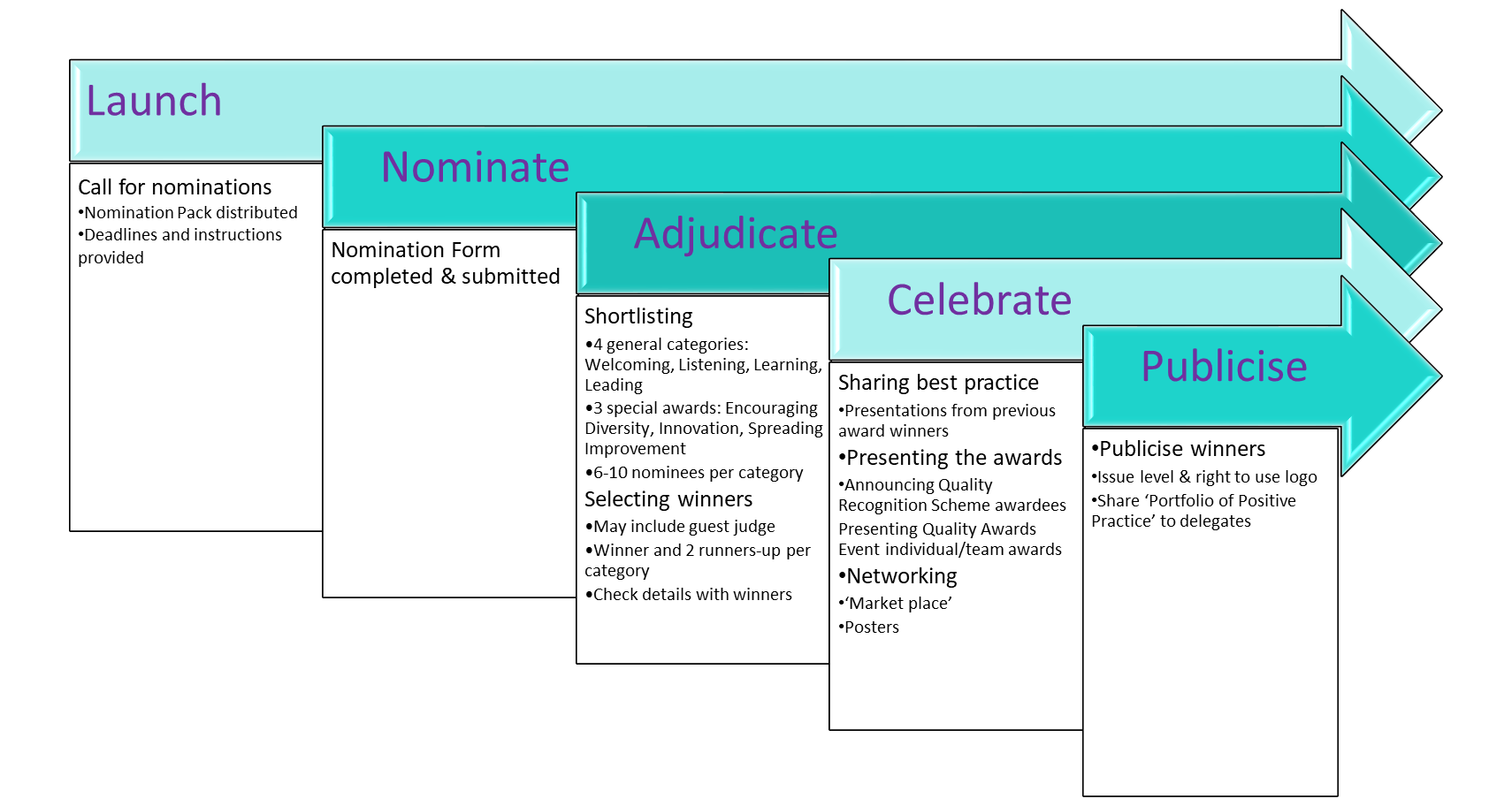


1. Expert Citizens National Insight Awards. <https://expertcitizens.org.uk/insightawards/> [↑](#footnote-ref-1)
