# Supplementary material for: The INSIGHT project: reflections on the co-production of a quality recognition programme to showcase excellence in public involvement in health and care research
Source: Res Involv Engagem. 2023 Oct 25;9:99. doi: 10.1186/s40900-023-00508-4 (PMC10601214; doi:10.1186/s40900-023-00508-4)
Supplement: Supplementary file 4 — Additional file 4. Bespoke online feedback questionnaire. [file 40900_2023_508_MOESM4_ESM.docx]

**ONLINE FEEDBACK QUESTIONNAIRE**

**SECTION 1: BACKGROUND INFORMATION**

1. Which of the following organisations are you affiliated to?

*Tick all that apply*

- - University Hospitals of North Midlands NHS Trust
  - Expert Citizens CIC/VOICES of Stoke
  - Keele University
  - Midlands Partnership Foundation Trust
  - Clinical Research Network West Midlands
  - Other (*please specify any others that are of relevance to the INSIGHT | Public Involvement project*) _____________________________________________

_______________________________________________________________

1. In what capacity you are affiliated to the organisations listed above:
   - Staff ___________________________________________________________

_______________________________________________________________

- - Public contributor ________________________________________________

_______________________________________________________________

- - Other (*please specify*) _____________________________________________

_______________________________________________________________

1. In which ways have you been involved in the project?

*Tick all that apply*

- - Was involved prior to the launch event
  - Attended the Launch event
  - As a member of Task & Finish Group 1 (Quality Standards)
  - As a member of Task & Finish Group 2 (Evaluation)
  - As a member of Task & Finish Group 3 (Training)
  - As a member of Task & Finish Group 4 (Awards Event)
  - As a member of the project Steering Group
  - As a member of the project Operational Group

**SECTION 2: THE PROJECT & YOU**

1. How clear to you was your role in the project?

*Tick one box*

- - Not clear at all
  - Not very clear
  - Somewhat clear
  - Quite clear
  - Completely clear

1. How satisfied were you with your role in the project?

*Tick one box*

- - Very dissatisfied
  - Somewhat dissatisfied
  - Neither satisfied nor dissatisfied
  - Somewhat satisfied
  - Very satisfied

1. How much do you feel you have made a contribution to the project personally?

*Tick one box*

- - None at all
  - A little
  - Some
  - Quite a lot
  - A great deal

1. Please describe how, if at all, you have made a contribution to the project personally.

________________________________________________________________________

________________________________________________________________________

________________________________________________________________________

________________________________________________________________________

________________________________________________________________________

1. How often you feel you were able to express your views freely about the project?

*Tick one box*

- - Never
  - Rarely
  - Sometimes
  - Often
  - Always

1. What, if anything, have you learned from the experience of being involved in the project?

________________________________________________________________________

________________________________________________________________________

________________________________________________________________________

________________________________________________________________________

________________________________________________________________________

**SECTION 3: ABOUT THE PROJECT**

1. How well do you feel you understand the overall objectives of the INSIGHT | Public Involvement project?

*Tick one box*

- - Did not understand anything
  - Understand a few things
  - Understand some things
  - Understand most things
  - Understand everything

1. How much impact do you think the INSIGHT | Public Involvement project could have on approaches to public involvement in health and care research?

*Tick one box*

- - None
  - Not much
  - A little
  - Quite a bit
  - A lot

1. How likely or unlikely will the INSIGHT | Public Involvement project be able to deliver this impact over the next 5 years?

*Tick one box*

- - Not likely at all
  - Somewhat unlikely
  - May or may not
  - Somewhat likely
  - Very likely

**SECTION 4: ABOUT YOU**

1. We would like to promote the role of public involvement in the INSIGHT | Public Involvement project. Are you happy for us to use your quotes on project-related reports, websites and promotional materials?

*Tick one box*

- - Yes
  - No

1. We would be interested in participating in the next phase of the INSIGHT | Public Involvement project (likely to be in 2022)?

*Tick one box*

- - Yes
  - No

1. Please use this space to explain any of your answers further or provide any other comments about your experience of being involved with the project?

________________________________________________________________________

________________________________________________________________________

________________________________________________________________________

________________________________________________________________________

________________________________________________________________________
